# Harnessing flagellin of *Ligilactobacillus agilis* as a surface display scaffold for an HIV-1 epitope

**DOI:** 10.1128/aem.00674-25

**Published:** 2025-05-29

**Authors:** Shunya Suzuki, Gregg A. Dean, Akinobu Kajikawa

**Affiliations:** 1 Department of Agricultural Chemistry, Tokyo University of Agriculture 13126, Setagaya, Tokyo, Japan; 2 Bioproduction Research Institute, National Institute of Advanced Industrial Science and Technology 267773 , Tsukuba, Ibaraki, Japan; 3 Department of Microbiology, Immunology and Pathology, College of Veterinary Medicine and Biomedical Sciences, Colorado State University 3447, Fort Collins, Colorado, USA; Universita degli Studi di Napoli Federico II, Portici, Italy

**Keywords:** flagellin, *Ligilactobacillus agilis*, lactic acid bacteria, TLR5, mucosal vaccine

## Abstract

Lactic acid bacteria (LAB) are promising mucosal vaccine vectors due to their safety, immunostimulatory properties, and the availability of genetic tools for certain strains. Bacterial flagellin has attracted attention as both a versatile scaffold for antigen surface display and a potent adjuvant via Toll-like receptor 5 (TLR5) activation. Despite these advantages, LAB-derived flagellin remains largely unexplored as an antigen display platform. In this study, we demonstrate the potential of flagellin (FliC2) from *Ligilactobacillus agilis*, one of the few flagellated LAB species, as a scaffold for the surface display of the HIV-1 membrane-proximal external region (MPER) epitope. Recombinant *L. agilis* strains were engineered to express FliC2 with the MPER epitope inserted at various positions within its hypervariable domain, identifying optimal sites for effective surface display. To enhance the adjuvant activity of FliC2, specific amino acid substitutions were introduced into the TLR5 recognition site, resulting in improved TLR5-stimulating activity *in vitro*. Immunization of mice with MPER-displaying *L. agilis* strains induced MPER-specific IgA and IgG responses, demonstrating the efficacy of the *L. agilis* flagellin-based display platform in eliciting both mucosal and systemic immune responses. This study is the first to demonstrate LAB-derived flagellin as an antigen display scaffold, highlighting *L. agilis* flagellin as a promising platform for mucosal vaccine development.

**IMPORTANCE:**

Lactic acid bacteria (LAB) are promising delivery vehicles of active molecules, and surface display systems are gaining interest for efficiently displaying heterologous peptides and proteins. Flagellin, a TLR5 agonist, has been widely used as an adjuvant and an antigen scaffold, making it a potentially valuable platform for such systems. However, the potential of LAB-derived flagellin as a surface display scaffold remains largely unexplored. This study demonstrates that flagellin from *Ligilactobacillus agilis*, a flagellated LAB species, effectively functions as an antigen display platform, eliciting mucosal and systemic immune responses. Our findings highlight the feasibility of LAB-derived flagellin as a versatile tool for surface display of heterologous peptides, expanding its potential applications in vaccine development and mucosal immunotherapy.

## INTRODUCTION

Mucosal vaccines represent a promising approach to combat infectious diseases and mucosal pathobionts-related diseases due to their ability to elicit both mucosal and systemic immune responses (1). Effective mucosal immune activation requires suitable vectors to deliver antigens to mucosal sites. Lactic acid bacteria (LAB) are attractive candidates for such vectors because of their safety, immunostimulatory properties, and the availability of genetic tools for certain strains (2). LAB are generally recognized as safe (GRAS) and widely used in food production and probiotics. Certain LAB strains exhibit immunostimulatory properties and can be genetically engineered to produce heterologous proteins and peptides. These properties make LAB promising candidates for mucosal vaccine vectors (2), and LAB-based mucosal vaccines against various pathogens have been investigated (3). Notably, a recent clinical trial of an oral vaccine based on *Lacticaseibacillus paracasei* expressing the HPV-16 E7 antigen demonstrated safety and efficacy in treating HPV-16-positive patients with cervical intraepithelial neoplasia (4), highlighting the potential of LAB-based mucosal vaccines. Despite these advances, the efficacies of mucosal vaccines, including LAB-based vaccines, are often insufficient, and innovative strategies to enhance their efficacy are required.

Bacterial flagellin is the major structural protein of flagella, produced by various bacteria, including a few LAB species. Flagellin has the potential to play dual roles in vaccine development as both an antigen display scaffold and an adjuvant. The flagellar filament comprises approximately 20,000 flagellin subunits, enabling the extensive display of antigens on the bacterial surface (5). Moreover, flagellin is recognized by Toll-like receptor 5 (TLR5), activating innate and adaptive immune responses, thereby serving as a potential adjuvant (6–8). These structural and immunological properties suggest that flagellin could play a crucial role in enhancing mucosal vaccine efficacy. Indeed, several studies have demonstrated the efficacy of flagellin derived from *Salmonella* as both an antigen display scaffold and an adjuvant. For example, recombinant *Salmonella* expressing flagellin displaying HIV-1 V3 loop epitopes successfully induced systemic and mucosal immune responses in mice (9). Similarly, Newton et al. reported strong antibody responses when cholera toxin epitopes were inserted into *Salmonella* flagellin (10). While these studies highlight the potential of flagellin from pathogenic bacteria in vaccine development, LAB-derived flagellin remains largely unexplored.

Most LAB lack flagella, but *Ligilactobacillus agilis* is one of the few flagellated species that inhabits the gastrointestinal tracts of various animals (11). Unlike typical LAB, *L. agilis* exhibits motility and chemotactic abilities, which may facilitate its penetration into the mucus layer and enhance survival under harsh conditions (12, 13). Moreover, *L. agilis* is genetically tractable (14), allowing for the production of heterologous proteins and targeted modifications of its flagellin (15, 16). Notably, *L. agilis* flagellin interacts with TLR5, and targeted amino acid substitutions can enhance its immunostimulatory activity (17). These capabilities suggest that *L. agilis* would be a versatile vector for mucosal vaccine development.

In this study, we harnessed the flagellin (FliC2) of *L. agilis* as a surface display scaffold for the HIV-1 membrane-proximal external region (MPER) epitope, a critical target for broadly neutralizing antibodies (18, 19). We engineered recombinant *L. agilis* strains to express FliC2 with the MPER epitope inserted at targeted positions within its hypervariable domain. To enhance immunogenicity, we introduced specific mutations into the TLR5 recognition site of FliC2 to improve its adjuvant activity. Through *in vitro* and *in vivo* analyses, we evaluated the surface display of the epitope, TLR5-stimulating activity, and immunogenic potential of these recombinant *L. agilis* strains. Our findings provide the first evidence of utilizing LAB-derived flagellin as an antigen display scaffold, establishing *L. agilis* flagellin as a promising platform for mucosal vaccine development.

## RESULTS AND DISCUSSION

*L. agilis* BKN88 possesses two active flagellin genes, *fliC1* and *fliC2*. Our previous study showed that *L. agilis* forms flagella and exhibits motility with either FliC1 or FliC2 alone (16). In addition, the FliC2-expressing strain exhibited higher immunological activity via TLR5 than the FliC1-expressing strain. Based on these findings, we selected *L. agilis* FliC2 as the scaffold for vaccine epitope display.

### Insertion of the MPER epitope into *L. agilis* FliC2

**Fig 1:**
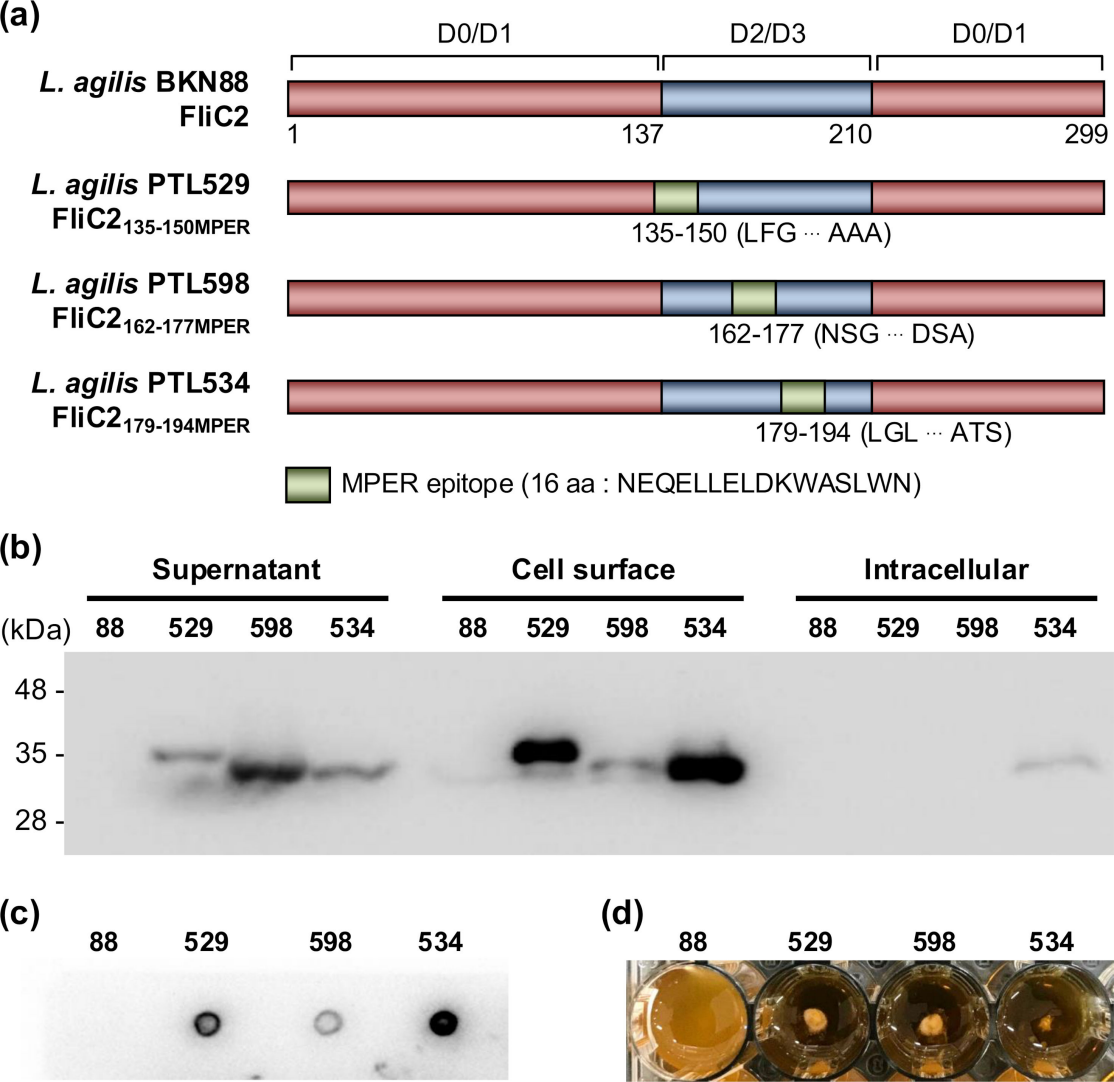
Construction and validation of recombinant *Ligilactobacillus agilis* strains expressing MPER-displaying flagellins. (a) Schematic representation of MPER-displaying flagellins. Domain architectures of FliC2 of *L. agilis* were predicted using InterPro. (b) Detection of the MPER epitope in FliC2 by Western blot using 2F5 mAb (anti-MPER monoclonal human IgG). Cell culture supernatant, cell surface, and intracellular proteins of *L. agilis* strains were analyzed by Western blot with 2F5 mAb. (c) Detection of the MPER epitope exposed on the cell surface of *L. agilis* strains by dot blot using 2F5 mAb. (d) The motility of *L. agilis* strains.

To determine optimal insertion sites for the MPER epitope within *L. agilis* FliC2, we constructed recombinant *L. agilis* strains expressing FliC2 variants with the MPER epitope (16 amino acid residues) inserted at different positions within its hypervariable domain. Structural analysis revealed that FliC2 consists of conserved D0/D1 domains and a hypervariable domain (Fig. 1a). Bacterial flagellins generally contain conserved D0/D1 domains and hypervariable D2/D3 domains (20), with the latter suggested to be not essential for TLR5-mediated immune activation (21). We thus replaced various regions of the hypervariable domain in *L. agilis* FliC2 with the MPER epitope and constructed recombinant *L. agilis* strains carrying each of the modified *fliC2* genes into the chromosome (Fig. 1a). Western blot analysis confirmed MPER-specific bands predominantly in the cell surface fraction of *L. agilis* strains PTL529 (expressing FliC2_135-150MPER_) and PTL534 (expressing FliC2_179-194MPER_) (Fig. 1b). In *L. agilis* PTL598 (expressing FliC2_162-177MPER_), the MPER-specific band was most prominent in the culture supernatant fraction, indicating that FliC2_162-177MPER_ is primarily secreted (Fig. 1b). In dot blot analysis, the MPER-specific signal was detected in all recombinant *L. agilis* strains, suggesting successful surface display of the MPER epitope (Fig. 1c). The signal intensity varied among the strains, and that of *L. agilis* PTL529 and PTL534 was clearly discernible. These results indicate that amino acid positions 135–150 and 179–194 within *L. agilis* FliC2 are suitable sites for epitope insertion and surface display.

Although PTL529 and PTL534 produced FliC2-MPER on the cell surface, neither strain formed flagella (Fig. S1) nor exhibited motility (Fig. 1d). Bacterial flagellin is secreted via a type III secretion system (T3SS) and assembled into flagella (22). It is possible that although the modified FliC2 is transported through T3SS, it fails to assemble into functional flagella. These results suggest that while the insertion of the MPER epitope into the FliC2 hypervariable domain impairs flagellar formation and motility, the modified FliC2 still effectively displays the epitope on the bacterial surface. Hence, due to its robust FliC2-MPER production, *L. agilis* PTL534 was selected for further analyses to evaluate its potential as a vaccine candidate.

### Construction and validation of a recombinant *L. agilis* expressing mutated FliC2 displaying MPER epitope

**Fig 2:**
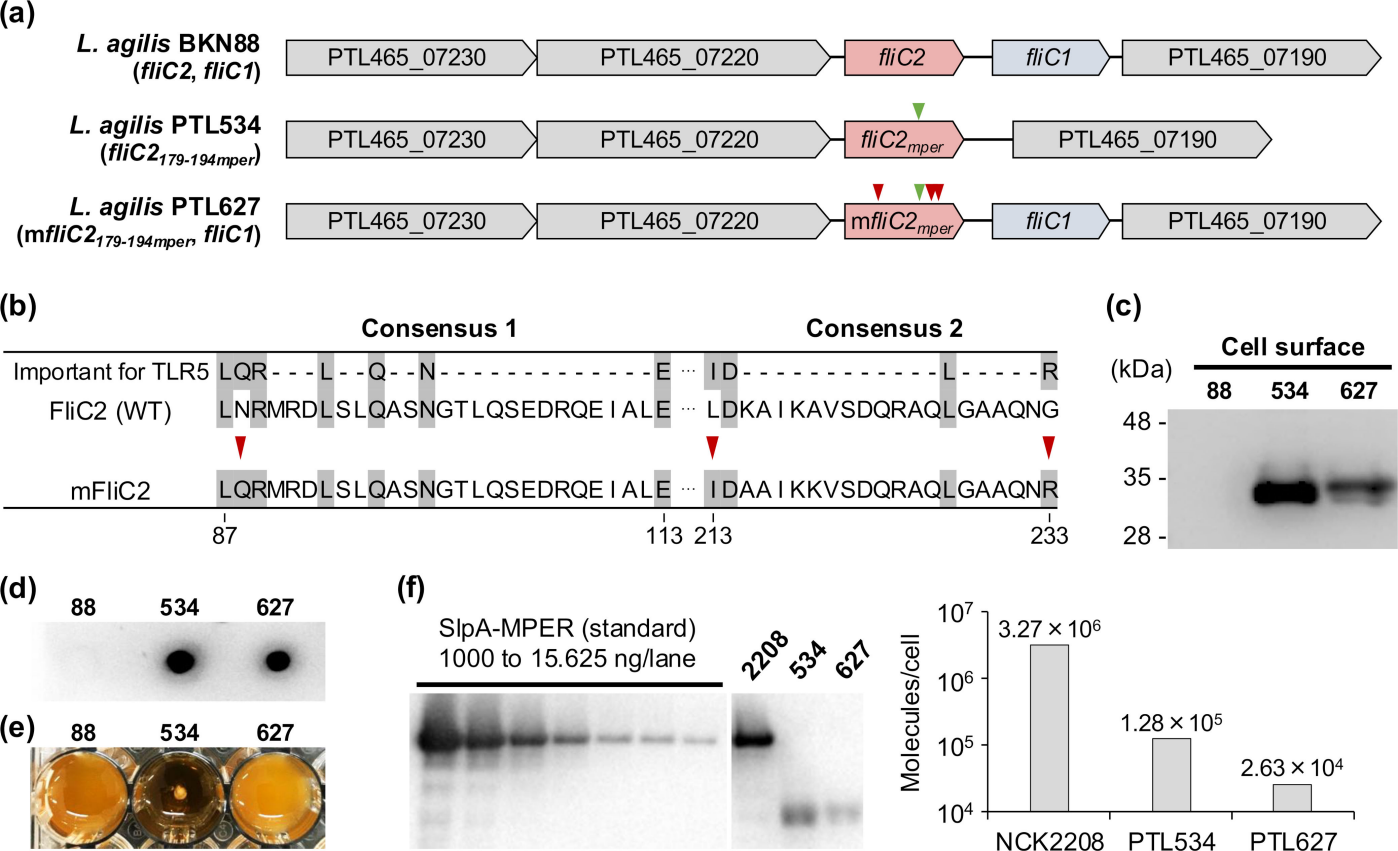
Construction and validation of a recombinant *L. agilis* expressing mutated FliC2 displaying MPER epitope. (a) Genetic map of the flagellin gene and flanking regions of *L. agilis* strains. The green and red arrowheads indicate epitope-replaced and *fliC2*-mutated sites, respectively. Locus tags are denoted in the arrows. (b) Alignment of amino acid sequences of FliC2 and mutated FliC2 (mFliC2). Conserved residues, which were reported to be important for TLR5 recognition, are highlighted in gray. The red arrowheads indicate the mutations in mFliC2. (c) Cell surface proteins of *L. agilis* strains were analyzed by Western blot with 2F5 mAb. (d) Detection of the MPER epitope exposed on the cell surface of *L. agilis* strains by dot blot using 2F5 mAb. (e) The motility of *L. agilis* strains. (f) Quantitative Western blot analysis of SlpA/FliC2 displaying MPER epitope on *Lactobacillus acidophilus* NCK2208/*L. agilis* strains. Left: *L. acidophilus* NCK2208 cells (1.75 × 10^6^ CFU/lane), *L. agilis* PTL534 or PTL627 cells (3.5 × 10^7^ CFU/lane), and the purified SlpA-MPER standards (1,000 to 15.625 ng/lane) were analyzed by Western blot with 2F5 mAb. Right: The MPER-specific signal intensity was quantified using ImageJ software, and the SlpA-MPER or FliC2-MPER molecules per cell were calculated.

To enhance the adjuvant activity of flagellin, targeted mutations were introduced into the FliC2-MPER of *L. agilis* PTL534 (Fig. 2a and b). Our previous study demonstrated that substituting three specific amino acids within the TLR5 recognition site of *L. agilis* FliC2 significantly increases its immunostimulatory activity (17). However, an *L. agilis* strain carrying only the mutated *fliC2* (m*fliC2*) could not produce mFliC2 on the cell surface. In contrast, when m*fliC2* was co-expressed with wild-type *fliC1*, the recombinant strain successfully produced the two flagellins on the surface and was more immunogenic than the wild-type *L. agilis* strain. Based on these findings, we constructed *L. agilis* PTL627 carrying both m*fliC2_179-194mper_* and *fliC1* genes on its chromosome (Fig. 2a and b). Western blot analysis confirmed the production of mFliC2-MPER on the bacterial surface (Fig. 2c), and dot blot analysis demonstrated successful exposure of the MPER epitope on the surface (Fig. 2d). Further phenotypic analyses revealed that PTL627 retained flagella (Fig. S1) and exhibited motility (Fig. 2e), unlike PTL534. These results showed that *L. agilis* PTL627 produces MPER-displaying FliC2 with the targeted mutations while maintaining flagellar formation and motility, making it a suitable candidate for further investigation.

Next, we estimated the amount of MPER-displaying flagellin in each bacterial cell using quantitative Western blot analysis with anti-MPER antibody (Fig. 2f; Fig. S2). The result showed that *L. agilis* PTL534 and PTL627 produced approximately 1.3 × 10^5^ and 2.6 × 10^4^ molecules of MPER-displaying flagellin per cell, respectively (Fig. 2f). The fivefold reduction in PTL627 compared with PTL534 may be attributed to the introduced mutations, which likely affect flagellar polymerization (20, 23, 24). Alternatively, the additional expression of *fliC1* might have affected flagellin production. Our analysis also estimated that *Lactobacillus acidophilus* NCK2208 (25), which produces MPER-displaying surface layer protein (SlpA-MPER), produced approximately 3.3 × 10^6^ molecules of SlpA-MPER per cell (Fig. 2f). Compared with *L. acidophilus* NCK2208, *L. agilis* PTL534 and PTL627 produced approximately 25- and 124-fold fewer MPER-displaying protein molecules, respectively. Nevertheless, the capacity to display epitopes of *L. agilis* flagellin is still comparable to that of other surface display systems in LAB, supporting its feasibility for vaccine development. For example, approximately 1.4 × 10^3^ to 3.9 × 10^3^ molecules per cell of tetanus toxin fragment C (TTFC) fused to a PrtP-derived LPXTG anchor have been displayed on the surface of *Lacticaseibacillus casei* (26). Other surface display systems, such as those based on LysM- and SLP-derived anchoring domains, have been reported to display approximately 4.5 × 10^3^ to 1.0 × 10^6^ and 1.1 × 10^6^ to 1.0 × 10^7^ molecules per cell, respectively (27–31). Collectively, these findings suggest that *L. agilis* FliC2 serves as an effective antigen display scaffold, while its expression levels vary depending on the presence of the targeted mutations and/or co-expression of FliC1.

### TLR5-mediated immunological activity of recombinant *L. agilis* strains

**Fig 3:**
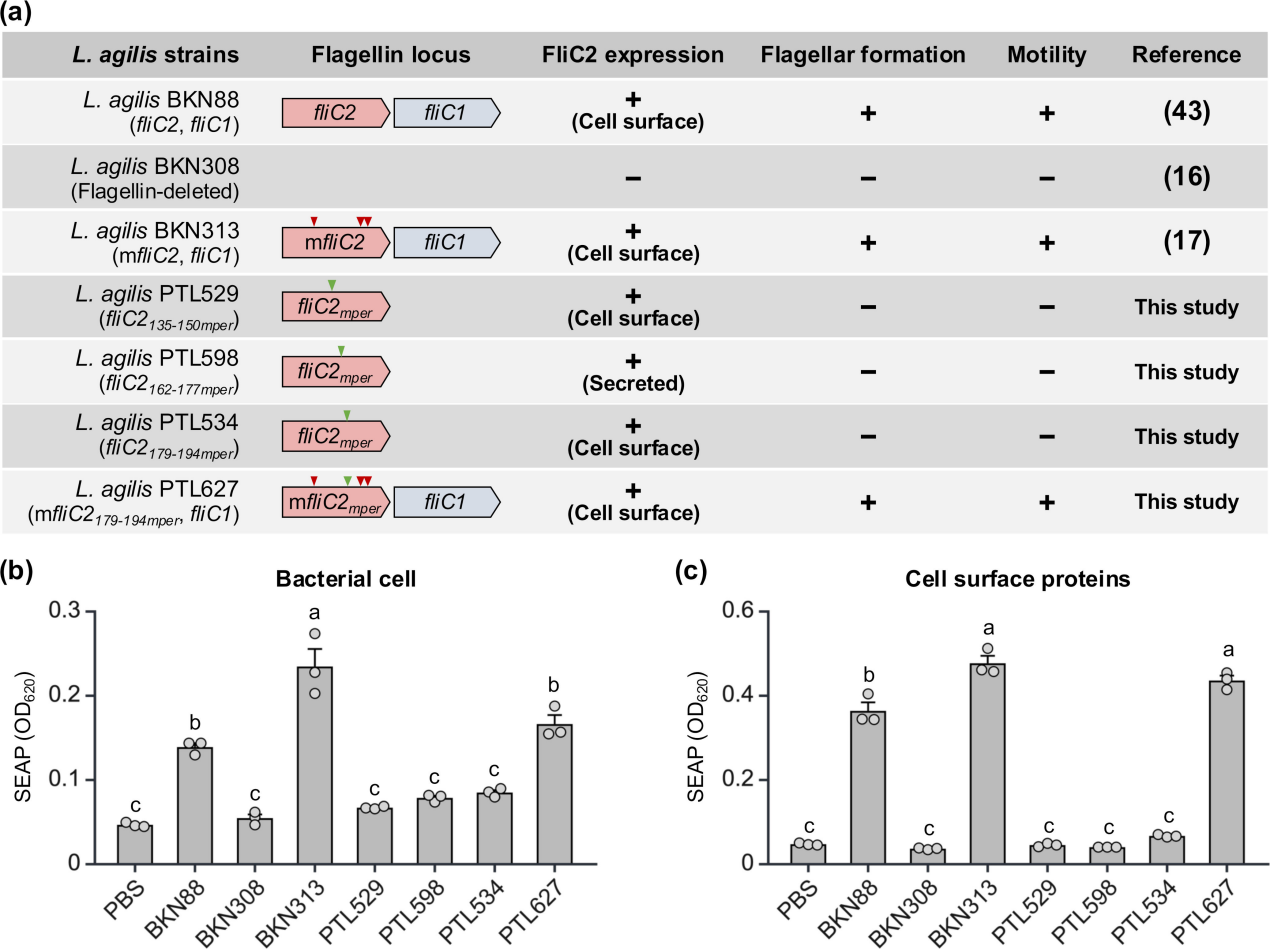
Immunological activity of recombinant *L. agilis* strains mediated via TLR5. (a) Genotypic and phenotypic characteristics of recombinant *L. agilis* strains. Partial genetic map of the flagellin gene of *L. agilis* strains is shown. The green and red arrowheads indicate epitope-replaced and fliC2 mutated sites, respectively. FliC2 expression was detected by either Western blot with 2F5 mAb or SDS-PAGE and CBB staining. Flagellar filaments were stained with FLAGELLA STAIN (Hardy Diagnostics) and observed using optical microscopy. The motility of *L. agilis* strains was observed using a semisolid MRS medium. (b and c) TLR5-reporter gene assay with whole *L. agilis* cells from different strains (b) and their cell surface proteins (c). Bacterial cells (1.0 × 10^7^ CFU/well) or cell surface proteins extracted from 1.0 × 10^7^ CFU of bacterial cells were added to HEK-Blue-hTLR5 cells (2.5 × 10^4^ cells/well), and released secreted alkaline phosphatase (SEAP) was measured. Values are mean plus standard error (*n* = 3). Statistical significance was determined by one-way ANOVA with Tukey multiple comparisons test. Different letters represent statistical significance.

The TLR5-stimulating activity of a series of *L. agilis* flagellin mutants (Fig. 3a) and their cell surface proteins was evaluated using a TLR5-reporter gene assay. As shown in Fig. 3b, BKN313 (expressing mFliC2 and FliC1) showed the highest TLR5 activation, while PTL529 (expressing FliC2_135-150MPER_), PTL598 (expressing FliC2_162-177MPER_), and PTL534 (expressing FliC2_179-194MPER_) exhibited little or no activity, similar to the flagellin-deficient strain BKN308. The activity of the wild-type strain BKN88 (expressing FliC2 and FliC1) and PTL627 (expressing mFliC2_179-194MPER_ and FliC1) was intermediate. A comparable pattern was observed in the TLR5-stimulating activity of the cell surface protein fractions, although the activity of PTL627 exhibited higher activity than that of BKN88 (Fig. 3c). Despite the production of FliC2-MPER in *L. agilis* PTL529, PTL598, and PTL534 (Fig. 1c and d), their TLR5-stimulating activity was as low as that of the flagellin-deleted strain BKN308. In contrast, our previous study showed that *L. agilis* expressing only FliC2 exhibited significantly higher TLR5-stimulating activity than BKN308 (16). These results indicate that the reduced activity of the FliC2-MPER-expressing strains is likely due to replacing the FliC2 hypervariable domain with the MPER epitope, rather than the absence of FliC1. Although several studies have demonstrated that the hypervariable domain is not essential for TLR5 activation (21, 32–35), Biedma et al. reported that deletion of this domain in *Salmonella* flagellin altered the secondary structure of the TLR5 recognition site, thereby reducing its activity via human TLR5 (hTLR5) but not mouse TLR5 (mTLR5) (36). Since our assays used HEK293 cells expressing hTLR5, it is possible that the MPER insertion affected the ability of FliC2 to activate hTLR5.

As expected, PTL627, which harbors both m*fliC2_179-194mper_* and *fliC1*, exhibited enhanced TLR5-stimulating activity, surpassing that of BKN88 (Fig. 3c). This increased activity may be attributed to the triple mutations introduced in the TLR5 recognition site of FliC2, as well as the additional expression of FliC1. Previous studies have shown that *L. agilis* expressing only FliC1 shows significantly lower TLR5 activation than that of BKN88 (16), supporting the idea that the mutations in FliC2 contributed to the enhanced TLR5 activation. As TLR5 activation by flagellin plays a critical role in promoting both innate and adaptive immune responses, *L. agilis* PTL627 may have potential as a vaccine candidate.

### Immunological potentials of recombinant *L. agilis* strains expressing MPER-displaying FliC2

**Fig 4:**
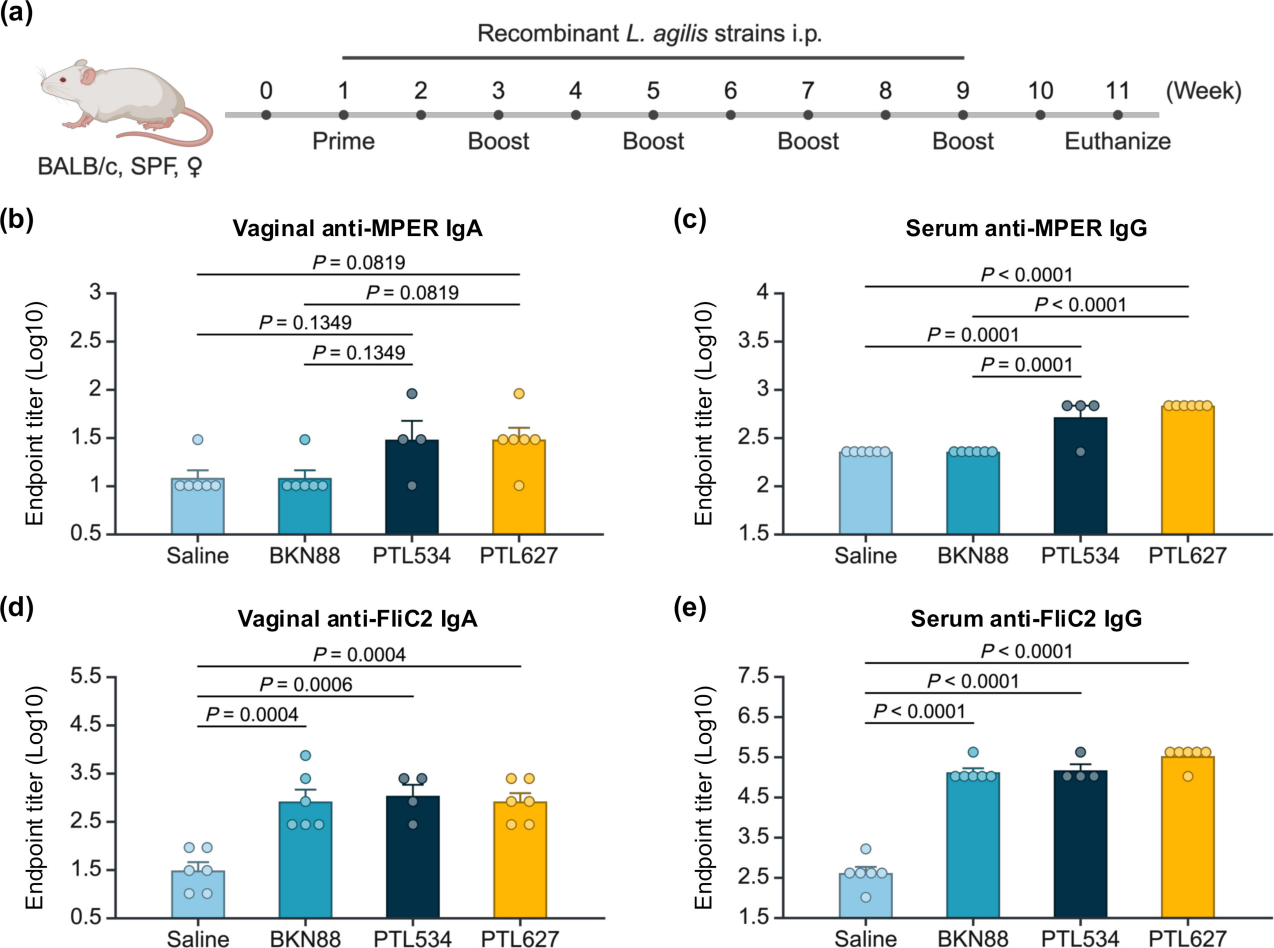
Immunological potentials of recombinant *L. agilis* strains expressing MPER-displaying flagellins. (a) Experimental schema. Mice were intraperitoneally immunized with either *L. agilis* BKN88, *L. agilis* PTL534, *L. agilis* PTL627, or sterile saline at 2 week intervals for a total of five immunizations (*n* = 4 to 6 mice/group). Two weeks after the last immunization, serum and vaginal washes were collected for quantification of antibody titers. (b and c) Endpoint titers of MPER-specific vaginal IgA (b) and serum IgG (c). (d and e) Endpoint titers of FliC2-specific vaginal IgA (d) and serum IgG (e). Values are mean plus standard error, and each dot represents an individual mouse. Statistical significance was determined by one-way ANOVA with Tukey multiple comparisons test, and *P*-values are shown.

To evaluate the immunological potential of MPER-displaying *L. agilis*, mice were immunized intraperitoneally with either *L. agilis* BKN88, PTL534, or PTL627, and the production of MPER- and FliC2-specific antibodies was measured (Fig. 4a). MPER-specific IgA titers in vaginal washes tended to increase in the PTL534 and PTL627 immunized groups compared with the BKN88 and saline groups, suggesting their ability to induce mucosal immune responses (Fig. 4b). In addition, immunization with PTL534 and PTL627 significantly elevated MPER-specific serum IgG titers, indicating a robust systemic antibody response against the epitope (Fig. 4c). These results demonstrate that the MPER epitope displayed on *L. agilis* flagellin can elicit both mucosal and systemic immune responses.

Endpoint titers of FliC2-specific IgA and IgG were comparable between all *L. agilis*-immunized groups and were significantly higher than those in the saline-immunized group (Fig. 4d and e). Interestingly, despite the differences in hTLR5-stimulating activity between PTL534 and PTL627, both strains elicited similar levels of MPER- and FliC2-specific antibodies. This discrepancy could be attributed to the approximately fivefold difference in FliC2-MPER production between PTL534 and PTL627, which may have influenced immune activation. Additionally, differences in flagellin recognition between human and mouse TLR5 could also account for the variation in immune activation. Previous studies have shown that mTLR5 is more tolerant of mutations and deletions in flagellin than hTLR5, which is more sensitive to structural changes of flagellin (36, 37). Thus, while *L. agilis* PTL534 exhibited minimal hTLR5-stimulating activity *in vitro*, it might still activate mTLR5, leading to comparable immune responses to PTL627 *in vivo*. However, there is no direct evidence to support this, and further studies are needed.

Collectively, these findings demonstrate that *L. agilis* flagellin is an effective antigen display platform, capable of eliciting both mucosal and systemic immune responses.

### Conclusion

This study demonstrates that *L. agilis* FliC2 can serve as both an antigen display scaffold and an immune activator. By inserting the MPER epitope into the hypervariable domain of FliC2, we successfully engineered *L. agilis* strains that effectively expose the epitope on their surface. Furthermore, targeted mutations in the TLR5 recognition site enhanced its immunostimulatory activity. Immunization with MPER-displaying *L. agilis* induced both mucosal and systemic MPER-specific antibody responses, underscoring its potential as a mucosal vaccine platform. This study provides the first evidence that *L. agilis* flagellin can function as an antigen delivery vector, offering a promising foundation for future mucosal vaccine development. However, since the immune responses in this study were evaluated following intraperitoneal immunization, further research is necessary to determine whether MPER-displaying *L. agilis* can elicit immune responses when administered through oral or intranasal routes. In addition, the roles of bacterial motility and TLR5-stimulating activity in antigen-specific immune induction remain to be elucidated. Despite the differences in motility and TLR5-stimulating activity between PTL534 and PTL627, both strains induced comparable antigen-specific immune responses. Further investigation into the impact of these factors—for example, through comparisons between PTL627 and an *L. agilis* mutant expressing FliC2-MPER and FliC1—could provide valuable insights for the rational design of more effective mucosal vaccines. Finally, while MPER-specific antibodies were successfully induced, their ability to neutralize HIV-1 remains to be determined. Future studies should investigate whether immunization with recombinant *L. agilis* confers protection against HIV-1 or other pathogens in relevant infection models. Such investigations will provide critical insights into the clinical applicability of this vaccine platform.

## MATERIALS AND METHODS

### Bacterial strains and growth conditions

The bacterial strains and plasmids used in this study are listed in Table 1. *L. agilis* BKN88, its derivative strains, and *L. acidophilus* were anaerobically propagated in MRS broth or agar (Difco, BD, USA) with or without 5 µg/mL of erythromycin at 37°C. *E. coli* EC101 and derivative strains were aerobically grown in Brain Heart Infusion (Difco, BD, USA) broth or agar with or without 200 µg/mL of erythromycin and 40 µg/mL of kanamycin at 37°C.

**TABLE 1:** Bacterial strains and plasmids used in this study

Strain or plasmid	Description and origin	Reference
*E. coli* strains		
EC101	Cloning host for pG^+^host5, RepA^+^	(38)
*Ligilactobacillus agilis* strains		
BKN88 (WT)	Isolated from avian gut, wild type	(39)
BKN308	Δ*fliC1*Δ*fliC2* (flagellin-deleted)	(16)
BKN310	Δ*fliC1* (*fliC2*-expressing)	(16)
BKN313	*fliC1* and mutated *fliC2* (m*fliC2*)-expressing	(17)
PTL529	*fliC2_135-150mper_* -expressing	This study
PTL534	*fliC2_179-194mper_* -expressing	This study
PTL598	*fliC2_162-177mper_* -expressing	This study
PTL627	*fliC1* and m*fliC2_179-194mper_* -expressing	This study
*Lactobacillus acidophilus* strain		
NCK2208	*slpA-mper*-expressing, NCFM derivative	(25)
Plasmids		
pG^+^host5	Replication-thermo-sensitive plasmid, Em^r^	(40)
pG^+^host5::m*fliC2, fliC1*	pG^+^host5 with mutated *fliC2* (m*fliC2*), *fliC1*, and flanking region	(17)
pG^+^host5::*fliC2*	pG^+^host5 with *fliC2* and flanking region	(16)

### Construction of recombinant *L. agilis* strains

Four kinds of recombinant *L. agilis* strains, *L. agilis* PTL529, PTL534, PTL598, and PTL627, were constructed by inserting *fliC2_135-150mper_*, *fliC2_179-194mper_*, *fliC2_162-177mper_*, or m*fliC2_179-194mper_*, and *fliC1* into the chromosome of the flagellin-deleted BKN308 strain of *L. agilis*, respectively. The plasmids and primers used for constructing the recombinant *L. agilis* strains are listed in Tables 1 and 2. The plasmids for inserting the modified flagellin gene were constructed from pG^+^host5::*fliC2* (16) or pG^+^host5::m*fliC2, fliC1* (17) using the in-Fusion HD Cloning Kit (Takara-bio, Shiga, Japan). The linearized plasmids were amplified by inverse PCR using primer pairs DOKJ797 and DOKJ798, DOKJ801 and DOKJ802, DOKJ1062 and DOKJ1063, or DOKJ1112 and DOKJ1113 with pG^+^host5::*fliC2* or pG^+^host5::m*fliC2, fliC1* as the template. MPER-encoding sequences were amplified by PCR using primer pairs DOKJ799 and DOKJ800, DOKJ803 and DOKJ804, DOKJ1064 and DOKJ1065, or DOKJ1114 and DOKJ1115. Chromosomal DNA of *L. acidophilus* NCK2208 was used as template. The linearized plasmid and the amplified MPER fragment were ligated using the In-Fusion HD Cloning Kit (Takara-bio, Shiga, Japan) and introduced into *E. coli* EC101. The transformants harboring plasmids with the desired insert were selected by colony PCR and Sanger sequencing using the M13 primers, DOKJ78 and DOKJ79. The constructed plasmids were introduced into *L. agilis* BKN308 by electroporation, and the modified flagellin gene was then inserted into the chromosome of BKN308 by double-crossing over, as described previously (16). The insertions of *fliC2_135-150mper_*, *fliC2_179-194mper_*, *fliC2_162-177mper_*, or m*fliC2_179-194mper_*, and *fliC1* genes in the constructed *L. agilis* strains, PTL529, PTL534, PTL598, and PTL627, were confirmed by PCR and Sanger sequencing using DOKJ705 and DOKJ706 primers.

**TABLE 2:** Primers used to construct recombinant *L. agilis* strains^*a*^

Primer	Sequence (5′ to 3′)
pG^+^host5::*fliC2_135-150mper_*	
DOKJ797	ACTTCAACTAGTTTTAAGTTCCAAGT
DOKJ798	ACCTAAGCCGTTAAACTTAGTG
DOKJ799	TTTAACGGCTTAGGTAACGAGCAAGAATTGTTAGA
DOKJ800	AAAACTAGTTGAAGTGTTCCACAAGCTAGCCCACT
pG^+^host5::*fliC2_162-177mper_*	
DOKJ1062	TCACTTGGTTTATACAGTGTGGC
DOKJ1063	AGCCCCTACTTGGAACTTAAAAC
DOKJ1064	TTCCAAGTAGGGGCTAACGAGCAAGAATTGTTAGAATTAGA
DOKJ1065	TGTATAAACCAAGTGAGTTCCACAAGCTAGCCCACT
pG^+^host5::*fliC2_179-194mper_*	
DOKJ801	GGTAAGTCAACTGCCTTTGCA
DOKJ802	TGAGGCTGAATCCATTGATTGG
DOKJ803	ATGGATTCAGCCTCAAACGAGCAAGAATTGTTAGA
DOKJ804	GGCAGTTGACTTACCGTTCCACAAGCTAGCCCACT
pG^+^host5::m*fliC2_179-194mper_*, *fliC1*	
DOKJ1112	ATCGATGTTAGAAACTAAAGTG
DOKJ1113	GCTTTAGGTAAGATAGATGCT
DOKJ1114	AGTTTCTAACATCGATGCTATCAGCAAGAAC
DOKJ1115	TATCTTACCTAAAGCAGTTGTCCCAGCAAATG
Flagellin flanking region	
DOKJ705	GGCTCATAGTTCATGCTAATGAAAAGTTAC
DOKJ706	GTCAGTAACTGGGAAGCAAACTCAAC
M13 primers	
DOKJ78	GTAAAACGACGGCCAGT
DOKJ79	CAGGAAACAGCTATGAC

^*a*^ Primers for inverse PCR are highlighted in gray.

### SDS-PAGE and Western blot

*L. agilis* strains were cultured overnight in MRS broth, and the overnight culture was centrifuged at 10,000 × *g* for 10 min at 4°C. The culture supernatant was precipitated with trichloroacetic acid (TCA), and the precipitated proteins were washed with acetone, followed by resuspension in 0.05 vol of 8 M urea. This suspension was used as cell culture supernatants. Cell surface proteins were extracted using 8 M urea. The bacterial cell pellet was washed twice with phosphate-buffered saline (PBS) and suspended in 0.05 vol of 8 M urea. Following vigorous homogenization, the bacterial cell suspension in 8 M urea was centrifuged at 10,000 × *g* for 10 min at 4°C, and the supernatant was collected as cell surface proteins. Intracellular proteins were prepared from the 8 M urea-treated cells with bead beating. The 8 M urea-treated cells were resuspended in 0.05 vol of 8 M urea, followed by bead beating with a FastPrep Instrument (MP Biomedicals, Santa Ana, CA, USA). Cellular debris was removed by centrifugation, and cell extracts were subsequently obtained as intracellular proteins. These samples were mixed with an equal volume of 2× Laemmli buffer (BIO-RAD, Hercules, CA, USA) containing 5% beta-mercaptoethanol and boiled for 5 min. The proteins were separated onto 5%–20% SDS-PAGE gels (c-PAGEL HR, ATTO, Tokyo, Japan), and the gels were transferred onto a PVDF membrane (Millipore, Burlington, MA, USA) for Western blotting. The membranes were blocked with 1% bovine serum albumin (BSA) in PBS containing 0.05% Tween 20 (PBST) for 1 h at room temperature with agitation and then incubated with 1% BSA in PBST containing the primary antibody (anti-HIV-1 gp41 monoclonal IgG, 2F5, which was obtained through the NIH AIDS Research and Reference Reagent Program, Division of AIDS, NIAID, NIH [Dr. Hermann Katinger]) for 1 h at room temperature with agitation. The membrane was washed thrice with PBST and incubated with 1% BSA in PBST containing horseradish-peroxidase (HRP)-conjugated anti-human IgG (Cell Signaling Technology Inc, Danvers, MA) for 1 h at room temperature with agitation. After the membrane was washed five times with PBST, MPER-specific signals were developed with Luminata Forte Western HRP Substrate (Millipore, USA) and visualized by ChemiDoc XRS + imaging system (Bio-Rad).

### Dot blot

*L. agilis* strains were cultured overnight in MRS broth, and the cells were harvested by centrifugation at 6,000 × *g* for 5 min at 4°C. The cell pellet was washed thrice with PBS and suspended in methanol. The whole-cell suspensions in methanol were then spotted on the PVDF membrane (Millipore, USA), and dried at room temperature for 20 min. The membrane was incubated with 1% BSA in PBS containing the primary antibody (anti-HIV-1 gp41 monoclonal IgG, 2F5) for 30 min at room temperature with agitation. The membrane was washed twice with PBS and incubated with 1% BSA in PBS containing the secondary antibody (HRP-conjugated anti-human IgG) for 30 min at room temperature with agitation. After the membrane was washed thrice with PBS, MPER-specific signals were developed with Luminata Forte Western HRP Substrate (Millipore, USA) and visualized by ChemiDoc XRS + imaging system (Bio-Rad).

### Isolation of flagellar filaments

The flagellar filaments were isolated as previously described (16). Briefly, bacterial cells in the mid-exponential phase were harvested and suspended in distilled water. The flagellar filaments were removed from the cells by homogenization using a FastPrep Instrument (MP Biomedicals, Santa Ana, CA, USA) at a speed setting of 6.5 m/s for 2 min. The cells were pelleted by centrifugation at 12,000 × *g* for 15 min at 4°C, and the supernatant containing the flagella was subsequently fractionated by ultracentrifugation at 100,000 × *g* for 1 h at 4°C. The precipitated flagella were suspended in a small amount of distilled water and stored at −20°C until use.

### Motility test and flagellar staining

The motility of *L. agilis* strains was observed using a semisolid MRS medium. *L. agilis* cells were inoculated into semisolid MRS medium containing 0.15% (w/v) agar and incubated at 37°C for 1 day. The cells that had migrated after growth in the semisolid MRS medium were observed. Bacterial motility in the mid-exponential phase was also observed using a BZ-X710 microscope (KEYENCE, Osaka, Japan). The flagellar filaments of *L. agilis* strains were stained with FLAGELLA STAIN (Hardy Diagnostics) according to the manufacturer’s protocol. The stained cells were subsequently visualized using a BZ-X710 microscope (KEYENCE, Osaka, Japan).

### *In silico* analysis

The GenBank accession number of *L. agilis* FliC2 is WP_172576007.1. Domain architectures of *L. agilis* FliC2 were analyzed with InterPro (41).

### Quantitative Western blot analysis

Extraction and purification of SlpA-MPER from *L. acidophilus* NCK2208 were performed with LiCl as described previously (42). The protein concentrations were determined using a Protein Assay Rapid kit wako II (Wako, Japan) according to the manufacturer’s protocol. The purified SlpA-MPER was used as a standard for quantitative western blotting. Bacterial cells in the exponential phase were harvested via centrifugation at 6,000 × *g* for 5 min at 4°C and gently washed twice with cold PBS. The cell pellet was suspended in PBS, then diluted and plated on MRS plates for enumeration. After centrifugation, the cell pellet was stored at −20°C until use. The bacterial cell pellet was suspended in 8 M urea and vigorously homogenized. The bacterial cell suspension and 2-fold serial dilution of the purified SlpA-MPER were mixed with an equal volume of 2 × Laemmli buffer (BIO-RAD, Hercules, CA, USA) containing 5% beta-mercaptoethanol and boiled for 5 min. *L. acidophilus* NCK2208 cells (1.75 × 10^6^ CFU/lane), *L. agilis* PTL534 or PTL627 cells (3.5 × 10^7^ CFU/lane), and the SlpA-MPER standards (1000 to 15.625 ng/lane) were loaded onto 5-20% SDS-PAGE gels (c-PAGEL HR, ATTO, Tokyo, Japan), and MPER-specific signals were detected by western blot with 2F5 mAb as described above. Western blot images were processed, and MPER-specific signal intensity was quantified using ImageJ software (National Institutes of Health, Bethesda, MD, USA), according to a previous study (43, 44). Subsequently, the SlpA-MPER or FliC2-MPER molecules per cell (CFU) were calculated from the acquired data. At least two independent experiments were performed.

### TLR5 reporter gene assay

Immunological activity via TLR5 was analyzed using HEK‐Blue‐hTLR5 cells (InvivoGen) according to the manufacturer’s instructions. Briefly, HEK-Blue-hTLR5 cells were maintained in Dulbecco’s modified Eagle medium (DMEM; Wako) supplemented with 10% (v/v) heat-inactivated fetal bovine serum (BD), 2 mM L-glutamine, 100 U/mL of penicillin (Gibco), 100 mg/mL of streptomycin (Gibco), 100 mg/mL of normocin (InvivoGen), 30 µg/mL of blasticidin (InvivoGen), and 100 µg/mL of zeocin (InvivoGen) in 5% CO2 at 37°C. Semi-confluent cultures of HEK-Blue-hTLR5 cells were harvested with cell scrapers. The cells were diluted with HEK-Blue Detection medium (InvivoGen) and then seeded to 96-well flat-bottom microplate wells (Thermo Fisher Scientific) at a concentration of 2.5 × 10^4^ cells/well. Bacteria cells from an overnight culture were harvested by centrifugation at 6,000 × *g* for 5 min at 4°C and washed three times with PBS, followed by resuspension in PBS. Cell surface proteins from the *L. agilis* cells were extracted by using 8 M urea. Bacterial cells were suspended in 8 M urea and vigorously homogenized. Following centrifugation at 10,000 × *g* for 10 min at 4°C, the supernatant was collected and diluted with PBS. Bacterial cells (1.0 × 10^7^ CFU/well) or cell surface proteins extracted from 1.0 × 10^7^ CFU of bacterial cells were added to each well and incubated in 5% CO_2_ at 37°C for 16 h. Secreted alkaline phosphatase (SEAP) production levels were determined by measuring the optical density at 620 nm (OD_620_) using an SH-1000 Lab Microplate Reader (Corona Electric).

### Immunization of mice

Female BALB/c mice were obtained from Crea Japan. Female BALB/c mice (10 weeks old) were intraperitoneally immunized with either *L. agilis* BKN88, *L. agilis* PTL534, *L. agilis* PTL627, or sterile saline. The *L. agilis* strains were cultured overnight in MRS broth, and the bacterial cells were collected by centrifugation at 6,000 × *g* for 5 min at 4°C. The cells were then washed thrice with PBS and suspended in sterile saline. Mice (4 to 6 mice/group) were immunized with 5.0 × 10^8^ CFU (for prime immunization) or 1.0 × 10^8^ CFU (for boost immunization) of the *L. agilis* strains, or saline at 2 week intervals for a total of 5 immunizations. Mice were euthanized 2 weeks after the last immunization, and blood was collected by cardiac puncture. The serum was separated by centrifugation at 3,000 × *g* for 10 min at 4°C. Vaginal lavage was collected by washing the vagina with PBS containing 0.05% Tween 20 using a pipet. Insoluble debris was removed by centrifugation at 10,000 × *g* for 10 min at 4°C. These samples were stored at −20°C until use.

### Enzyme-linked immunosorbent assay (ELISA) for quantification of antibody titers

Ninety-six-well flat-bottom microplate wells (Thermo Fisher Scientific) were coated with either 1 µg/mL of synthetic MPER peptide (Bio-Synthesis Inc, Lewisville, TX) or 5 µg/mL of purified FliC2 isolated from *L. agilis* BKN310 cells in carbonate buffer (15 mM Na_2_CO_3_ and 35 mM NaHCO_3_; pH 9.6) overnight at 4°C. After washing three times with PBST, the wells were blocked with 1% BSA in PBS for 1 h at room temperature and washed three times with PBST. Samples were serially diluted with 1% BSA in PBST and added to each well, followed by incubation for 2 h at room temperature. Following washing five times with PBST, the wells were subsequently incubated with either HRP-conjugated anti-mouse IgG (A2304, SIGMA) for the serum samples or HRP-conjugated anti-mouse IgA (ab97235, abcam) for the vaginal samples. After incubation for 1 h at room temperature, the wells were washed seven times with PBST, and the color was developed by incubating with 3,3′,5,5′-tetramethylbenzidine (TMB) for 30 min at room temperature. The reaction was stopped with 2N H_2_SO_4_, and the absorbance was measured at 450–570 nm using an SH-1000 Lab Microplate Reader (Corona Electric). The mean value plus 3 standard deviations (SD) of the samples from the saline-immunized group was applied as the cut-off value to determine the endpoint titer.

## SUPPLEMENTAL MATERIAL

Supplemental figures aem.00674-25-s0001.pdf Fig. S1 and S2.

ASM does not own the copyrights to Supplemental Material that may be linked to, or accessed through, an article. The authors have granted ASM a non-exclusive, world-wide license to publish the Supplemental Material files. Please contact the corresponding author directly for reuse.

## References

[1] Lavelle EC, Ward RW. 2022. Mucosal vaccines - fortifying the frontiers. Nat Rev Immunol 22:236–250. doi:10.1038/s41577-021-00583-2 34312520 PMC8312369

[2] Wells JM, Mercenier A. 2008. Mucosal delivery of therapeutic and prophylactic molecules using lactic acid bacteria. Nat Rev Microbiol 6:349–362. doi:10.1038/nrmicro1840 18345021 PMC7096801

[3] LeCureux JS, Dean GA. 2018. Lactobacillus mucosal vaccine vectors: immune responses against bacterial and viral antigens. mSphere 3:e00061-18. doi:10.1128/mSphere.00061-18 29769376 PMC5956152

[4] Kawana K, Kobayashi O, Ikeda Y, Yahata H, Iwata T, Satoh T, Akiyama A, Maeda D, Hori-Hirose Y, Uemura Y, Nakayama-Hosoya K, Katoh K, Katoh Y, Nakajima T, Taguchi A, Komatsu A, Asai-Sato M, Tomita N, Kato K, Aoki D, Igimi S, Kawana-Tachikawa A, Schust DJ. 2023. Phase I and II randomized clinical trial of an oral therapeutic vaccine targeting human papillomavirus for treatment of cervical intraepithelial neoplasia 2 and 3. JNCI Cancer Spectr 7. doi:10.1093/jncics/pkad101 PMC10748578 38001029

[5] Westerlund-Wikström B. 2000. Peptide display on bacterial flagella: principles and applications. Int J Med Microbiol 290:223–230. doi:10.1016/S1438-4221(00)80119-8 10959724

[6] Cui B, Liu X, Fang Y, Zhou P, Zhang Y, Wang Y. 2018. Flagellin as a vaccine adjuvant. Expert Rev Vaccines 17:335–349. doi:10.1080/14760584.2018.1457443 29580106

[7] Mizel SB, Bates JT. 2010. Flagellin as an adjuvant: cellular mechanisms and potential. J Immunol 185:5677–5682. doi:10.4049/jimmunol.1002156 21048152 PMC3756556

[8] Hajam IA, Dar PA, Shahnawaz I, Jaume JC, Lee JH. 2017. Bacterial flagellin-a potent immunomodulatory agent. Exp Mol Med 49:e373. doi:10.1038/emm.2017.172 28860663 PMC5628280

[9] Cattozzo EM, Stocker BA, Radaelli A, De Giuli Morghen C, Tognon M. 1997. Expression and immunogenicity of V3 loop epitopes of HIV-1, isolates SC and WMJ2, inserted in Salmonella flagellin. J Biotechnol 56:191–203. doi:10.1016/s0168-1656(97)00117-x 9304878

[10] Newton SMC, Jacob CO, Stocker BAD. 1989. Immune response to cholera toxin epitope inserted in Salmonella flagellin. Science 244:70–72. doi:10.1126/science.2468182 2468182

[11] Suzuki S, Fujita K, Maeno S, Shiwa Y, Endo A, Yokota K, Igimi S, Kajikawa A. 2020. PCR-based screening, isolation, and partial characterization of motile lactobacilli from various animal feces. BMC Microbiol 20:142. doi:10.1186/s12866-020-01830-7 32493209 PMC7268542

[12] Kajikawa A, Suzuki S, Igimi S. 2018. The impact of motility on the localization of Lactobacillus agilis in the murine gastrointestinal tract. BMC Microbiol 18:68. doi:10.1186/s12866-018-1219-3 29996774 PMC6042280

[13] Suzuki S, Yokota K, Igimi S, Kajikawa A. 2023. Negative chemotaxis of Ligilactobacillus agilis BKN88 against gut-derived substances. Sci Rep 13:15632. doi:10.1038/s41598-023-42840-5 37730901 PMC10511705

[14] Stephenson DP, Moore RJ, Allison GE. 2011. Transformation of, and heterologous protein expression in, Lactobacillus agilis and Lactobacillus vaginalis isolates from the chicken gastrointestinal tract. Appl Environ Microbiol 77:220–228. doi:10.1128/AEM.02006-10 21075881 PMC3019714

[15] Vezina B, Allnutt T, Keyburn AL, Wade B, Van TTH, Johanesen P, Lyras D, Moore RJ. 2021. Stable recombinant-gene expression from a Ligilactobacillus live bacterial vector via chromosomal integration. Appl Environ Microbiol 87:e00392-21. doi:10.1128/AEM.00392-21 33741626 PMC8208147

[16] Eguchi N, Suzuki S, Yokota K, Igimi S, Kajikawa A. 2021. Ligilactobacillus agilis BKN88 possesses thermo-/acid-stable heteropolymeric flagellar filaments. Microbiology (Reading) 167. doi:10.1099/mic.0.001020 33502302

[17] Kajikawa A, Eguchi N, Suzuki S. 2022. Immunogenic modification of Ligilactobacillus agilis by specific amino acid substitution of flagellin. Appl Environ Microbiol 88:e0127722. doi:10.1128/aem.01277-22 36173204 PMC9599256

[18] Williams WB, Alam SM, Ofek G, Erdmann N, Montefiori DC, Seaman MS, Wagh K, Korber B, Edwards RJ, Mansouri K. 2024. Vaccine induction of heterologous HIV-1-neutralizing antibody B cell lineages in humans. Cell 187:2919–2934. doi:10.1016/j.cell.2024.04.033 38761800 PMC11993910

[19] López CA, Alam SM, Derdeyn CA, Haynes BF, Gnanakaran S. 2024. Influence of membrane on the antigen presentation of the HIV-1 envelope membrane proximal external region (MPER). Curr Opin Struct Biol 88:102897. doi:10.1016/j.sbi.2024.102897 39173417

[20] Samatey FA, Imada K, Nagashima S, Vonderviszt F, Kumasaka T, Yamamoto M, Namba K. 2001. Structure of the bacterial flagellar protofilament and implications for a switch for supercoiling. Nature 410:331–337. doi:10.1038/35066504 11268201

[21] Afzal H, Murtaza A, Cheng LT. 2025. Structural engineering of flagellin as vaccine adjuvant: quest for the minimal domain of flagellin for TLR5 activation. Mol Biol Rep 52:104. doi:10.1007/s11033-024-10146-y 39775323 PMC11706886

[22] Diepold A, Armitage JP. 2015. Type III secretion systems: the bacterial flagellum and the injectisome. Philos Trans R Soc Lond B Biol Sci 370:20150020. doi:10.1098/rstb.2015.0020 26370933 PMC4632597

[23] Smith KD, Andersen-Nissen E, Hayashi F, Strobe K, Bergman MA, Barrett SLR, Cookson BT, Aderem A. 2003. Toll-like receptor 5 recognizes a conserved site on flagellin required for protofilament formation and bacterial motility. Nat Immunol 4:1247–1253. doi:10.1038/ni1011 14625549

[24] Yoon S, Kurnasov O, Natarajan V, Hong M, Gudkov AV, Osterman AL, Wilson IA. 2012. Structural basis of TLR5-flagellin recognition and signaling. Science 335:859–864. doi:10.1126/science.1215584 22344444 PMC3406927

[25] Kajikawa A, Zhang L, LaVoy A, Bumgardner S, Klaenhammer TR, Dean GA. 2015. Mucosal immunogenicity of genetically modified Lactobacillus acidophilus expressing an HIV-1 epitope within the surface layer protein. Plos One 10:e0141713. doi:10.1371/journal.pone.0141713 26509697 PMC4624987

[26] Maassen CB, Laman JD, den Bak-Glashouwer MJ, Tielen FJ, van Holten-Neelen JC, Hoogteijling L, Antonissen C, Leer RJ, Pouwels PH, Boersma WJ, Shaw DM. 1999. Instruments for oral disease-intervention strategies: recombinant Lactobacillus casei expressing tetanus toxin fragment C for vaccination or myelin proteins for oral tolerance induction in multiple sclerosis. Vaccine (Auckl) 17:2117–2128. doi:10.1016/s0264-410x(99)00010-9 10367944

[27] Bosma T, Kanninga R, Neef J, Audouy SAL, van Roosmalen ML, Steen A, Buist G, Kok J, Kuipers OP, Robillard G, Leenhouts K. 2006. Novel surface display system for proteins on non-genetically modified gram-positive bacteria. Appl Environ Microbiol 72:880–889. doi:10.1128/AEM.72.1.880-889.2006 16391130 PMC1352190

[28] Ravnikar M, Štrukelj B, Obermajer N, Lunder M, Berlec A. 2010. Engineered lactic acid bacterium. Appl Environ Microbiol 76:6928–6932. doi:10.1128/AEM.00190-10 20802083 PMC2953040

[29] Hu S, Kong J, Kong W, Guo T, Ji M. 2010. Characterization of a novel LysM domain from Lactobacillus fermentum bacteriophage endolysin and its use as an anchor to display heterologous proteins on the surfaces of lactic acid bacteria. Appl Environ Microbiol 76:2410–2418. doi:10.1128/AEM.01752-09 20173067 PMC2849211

[30] Hu S, Kong J, Sun Z, Han L, Kong W, Yang P. 2011. Heterologous protein display on the cell surface of lactic acid bacteria mediated by the s-layer protein. Microb Cell Fact 10:86. doi:10.1186/1475-2859-10-86 22035337 PMC3215925

[31] Gordillo TB, Palumbo MC, Allievi MC, Fernández Do Porto DA, Ruzal SM, Palomino MM. 2020. Strategies to display heterologous proteins on the cell surface of lactic acid bacteria using as anchor the C-terminal domain of Lactobacillus acidophilus SlpA. World J Microbiol Biotechnol 36:169. doi:10.1007/s11274-020-02945-9 33043388

[32] Yang J, Zhong M, Zhang Y, Zhang E, Sun Y, Cao Y, Li Y, Zhou D, He B, Chen Y, Yang Y, Yu J, Yan H. 2013. Antigen replacement of domains D2 and D3 in flagellin promotes mucosal IgA production and attenuates flagellin-induced inflammatory response after intranasal immunization. Hum Vaccin Immunother 9:1084–1092. doi:10.4161/hv.23809 23377752 PMC3899144

[33] Khim K, Bang YJ, Puth S, Choi Y, Lee YS, Jeong K, Lee SE, Rhee JH. 2021. Deimmunization of flagellin for repeated administration as a vaccine adjuvant. NPJ Vaccines 6:116. doi:10.1038/s41541-021-00379-4 34518537 PMC8438039

[34] Rhee JH, Khim K, Puth S, Choi Y, Lee SE. 2023. Deimmunization of flagellin adjuvant for clinical application. Curr Opin Virol 60:101330. doi:10.1016/j.coviro.2023.101330 37084463

[35] Nempont C, Cayet D, Rumbo M, Bompard C, Villeret V, Sirard J-C. 2008. Deletion of flagellin’s hypervariable region abrogates antibody-mediated neutralization and systemic activation of TLR5-dependent immunity. J Immunol 181:2036–2043. doi:10.4049/jimmunol.181.3.2036 18641341

[36] Biedma ME, Cayet D, Tabareau J, Rossi AH, Ivičak-Kocjan K, Moreno G, Errea A, Soulard D, Parisi G, Jerala R, Berguer P, Rumbo M, Sirard JC. 2019. Recombinant flagellins with deletions in domains D1, D2, and D3: characterization as novel immunoadjuvants. Vaccine (Auckl) 37:652–663. doi:10.1016/j.vaccine.2018.12.009 30583910

[37] Forstnerič V, Ivičak-Kocjan K, Ljubetič A, Jerala R, Benčina M. 2016. Distinctive recognition of flagellin by human and mouse toll-like receptor 5. Plos One 11:e0158894. doi:10.1371/journal.pone.0158894 27391968 PMC4938411

[38] Law J, Buist G, Haandrikman A, Kok J, Venema G, Leenhouts K. 1995. A system to generate chromosomal mutations in Lactococcus lactis which allows fast analysis of targeted genes. J Bacteriol 177:7011–7018. doi:10.1128/jb.177.24.7011-7018.1995 8522504 PMC177576

[39] Kajikawa A, Midorikawa E, Masuda K, Kondo K, Irisawa T, Igimi S, Okada S. 2016. Characterization of flagellins isolated from a highly motile strain of Lactobacillus agilis. BMC Microbiol 16:49. doi:10.1186/s12866-016-0667-x 27001290 PMC4802830

[40] Biswas I, Gruss A, Ehrlich SD, Maguin E. 1993. High-efficiency gene inactivation and replacement system for gram-positive bacteria. J Bacteriol 175:3628–3635. doi:10.1128/jb.175.11.3628-3635.1993 8501066 PMC204764

[41] Blum M, Andreeva A, Florentino LC, Chuguransky SR, Grego T, Hobbs E, Pinto BL. 2025. InterPro: the protein sequence classification resource in 2025. Nucleic Acids Res 53:D444–D456. doi:10.1093/nar/gkae1082 39565202 PMC11701551

[42] Suzuki S, Yokota K, Igimi S, Kajikawa A. 2019. Comparative analysis of immunological properties of S-layer proteins isolated from Lactobacillus strains. Microbiology (Reading) 165:188–196. doi:10.1099/mic.0.000766 30620267

[43] Pillai-Kastoori L, Schutz-Geschwender AR, Harford JA. 2020. A systematic approach to quantitative Western blot analysis. Anal Biochem 593:113608. doi:10.1016/j.ab.2020.113608 32007473

[44] Gallo-Oller G, Ordoñez R, Dotor J. 2018. A new background subtraction method for western blot densitometry band quantification through image analysis software. J Immunol Methods 457:1–5. doi:10.1016/j.jim.2018.03.004 29522776

