# Supplementary material for: Harnessing flagellin of Ligilactobacillus agilis as a surface display scaffold for an HIV-1 epitope
Source: Appl Environ Microbiol. 2025 May 29;91(6):e00674-25. doi: 10.1128/aem.00674-25 (PMC12175526; doi:10.1128/aem.00674-25)
Supplement: Supplemental figures — Fig. S1 and S2. [file aem.00674-25-s0001.pdf]

Supplemental Information

Harnessing flagellin of *Ligilactobacillus agilis* as a surface display scaffold for an HIV-1 Epitope

Shunya Suzuki [a,b], Gregg A. Dean [c], and Akinobu Kajikawa [a*]

[a] Department of Agricultural Chemistry, Graduate school of Tokyo University of Agriculture, 1-1-1 Sakuragaoka, Setagaya, Tokyo 156-8502, Japan.
[b] Bioproduction Research Institute, National Institute of Advanced Industrial Science and Technology, 1-1-1 Higashi, Tsukuba, Ibaraki 305-8566, Japan
[c] Department of Microbiology, Immunology and Pathology, College of Veterinary Medicine and Biomedical Sciences, Colorado State University, Fort Collins, CO 80523, USA

***Corresponding author**
Akinobu Kajikawa, a3kajika@nodai.ac.jp

This file includes

- Supplementary Figures S1 to S2

Supplemental Figures

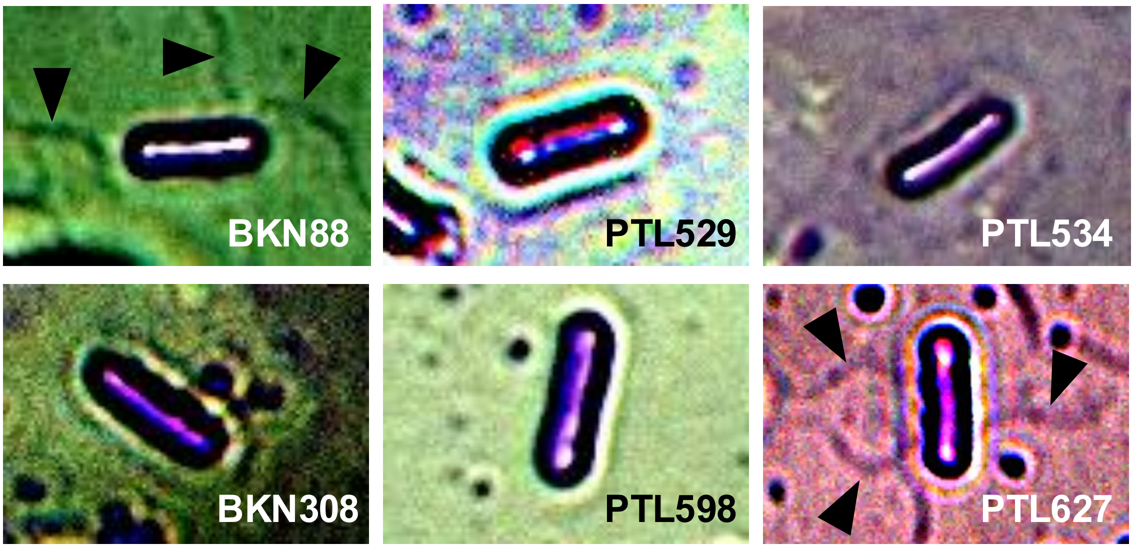

Figure S1. Flagella staining of *L. agilis* strains. The flagellar filaments of *L. agilis* BKN88 (wild-type), BKN308 (Δ*fliC1*Δ*fliC2*), PTL529 ($fliC2_{135\text{-}150mper}$–expressing), PTL598 ($fliC2_{162\text{-}177mper}$–expressing), PTL534 ($fliC2_{179\text{-}194mper}$–expressing), and PTL627 (*fliC1* and $fliC2_{179\text{-}194mper}$–expressing) were stained with FLAGELLA STAIN (Hardy Diagnostics). The stained flagella were observed using optical microscopy. The arrowheads represent the flagellar filaments.

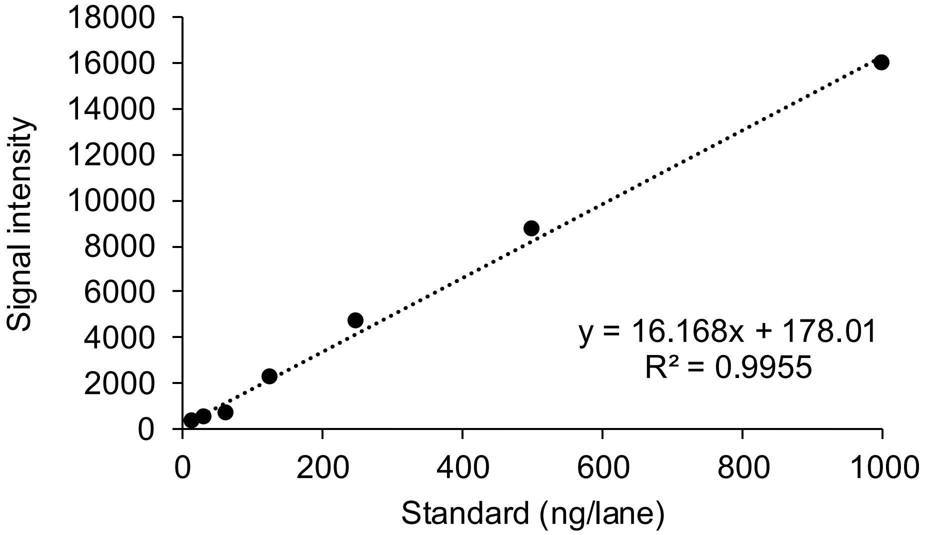

Figure S2. Standard curve of signal intensity versus total protein loaded. The purified SlpA-MPER standards (1000 to 15.625 ng/lane) were loaded onto SDS-PAGE gels, and MPER-specific signals were detected by western blot with 2F5 mAb. The signal intensity was quantified and plotted using ImageJ software.
